# Oxidative Stress/Angiotensinogen/Renin-Angiotensin System Axis in Patients with Diabetic Nephropathy

**DOI:** 10.3390/ijms141123045

**Published:** 2013-11-21

**Authors:** Masumi Kamiyama, Maki Urushihara, Takashi Morikawa, Yoshio Konishi, Masahito Imanishi, Akira Nishiyama, Hiroyuki Kobori

**Affiliations:** 1 Department of Physiology, Tulane University Health Sciences Center, 1430 Tulane Avenue, New Orleans, LA 70112, USA; E-Mails: kamiyama1206@gmail.com (M.K.); murushih@tulane.edu (M.U.); 2 Hypertension and Renal Center of Excellence, Tulane University Health Sciences Center, 1430 Tulane Avenue, New Orleans, LA 70112, USA; 3 Department of Nephrology and Hypertension, Osaka City General Hospital, 2-13-22 Miyakojima-Hondori, Miyakojima-ku, Osaka 534-0021, Japan; E-Mails: konitiha@rr.iij4u.or.jp (T.M.); ymsry-ko@msic.med.osaka-cu.ac.jp (Y.K.); masachan@msic.med.osaka-cu.ac.jp (M.I.); 4 Department of Pharmacology, Kagawa University Medical School, Miki, 1750-1 Ikenobe, Miki, Kagawa 761-0793, Japan; E-Mail: akira@med.kagawa-u.ac.jp; 5 Department of Medicine, Tulane University Health Sciences Center, 1430 Tulane Avenue, New Orleans, LA 70112, USA

**Keywords:** angiotensinogen, clinical study, diabetic nephropathy, oxidative stress, renin-angiotensin system

## Abstract

Although recent studies have proven that renin-angiotensin system (RAS) blockades retard the progression of diabetic nephropathy, the detailed mechanisms of their reno-protective effects on the development of diabetic nephropathy remain uncertain. In rodent models, it has been reported that reactive oxygen species (ROS) are important for intrarenal angiotensinogen (AGT) augmentation in the progression of diabetic nephropathy. However, no direct evidence is available to demonstrate that AGT expression is enhanced in the kidneys of patients with diabetes. To examine whether the expression levels of ROS- and RAS-related factors in kidneys are increased with the progression of diabetic nephropathy, biopsied samples from 8 controls and 27 patients with type 2 diabetes were used. After the biopsy, these patients were diagnosed with minor glomerular abnormality or diabetes mellitus by clinical and pathological findings. The intensities of AGT, angiotensin II (Ang II), 4-hydroxy-2-nonenal (4-HNE), and heme oxygenase-1 (HO-1) were examined by fluorescence *in situ* hybridization and/or immunohistochemistry. Expression levels were greater in patients with diabetes than in control subjects. Moreover, the augmented intrarenal AGT mRNA expression paralleled renal dysfunction in patients with diabetes. These data suggest the importance of the activated oxidative stress/AGT/RAS axis in the pathogenesis of diabetic nephropathy.

## 1. Introduction

Activation of the renin-angiotensin system (RAS) is thought to be the major mechanism underlying diabetic nephropathy. Several reports have demonstrated that high glucose levels are the principal cause of renal damage in both type 1 [1] and type 2 [2] diabetes. Other studies have reported that various renal cell types cultured in a high glucose medium or stimulated by high levels of glucose exhibit cellular hypertrophy, cell proliferation, and excessive production of extracellular matrix, which are typical features of diabetic nephropathy [3,4].

While the mechanisms underlying the development of diabetic nephropathy are extremely complex [5], the activation of the intrarenal RAS has been suggested as a possible mechanism [6–8]. The renoprotective effect of the RAS blockade in patients with diabetes was first reported over 25 years ago [9]. Since then, it has been reported that both angiotensin converting enzyme (ACE) inhibitors and angiotensin II receptor blockers (ARBs) delay the development and progression of diabetic nephropathy [10–12]. Currently, the detailed mechanisms of the development of diabetic nephropathy remain uncertain.

Angiotensinogen (AGT) is the only known substrate for renin, which is the rate-limiting enzyme of the RAS [13]. Because the level of AGT is close to the Michaelis-Menten constant for renin, both renin and AGT levels can control the activity of the RAS. In particular, upregulation of AGT levels lead to elevated angiotensin peptide levels [14,15]. It has been shown that renal AGT is predominantly localized in the proximal tubules [16–18], whereas weak expression is detected in glomeruli [19], and that high glucose levels stimulate AGT expression in kidney cells [20–22]. Other authors and ourselves have also reported the augmentation of intrarenal AGT expression in streptozotocin-induced type 1 diabetic mice [23] and Zucker Diabetic Fatty (ZDF) type 2 diabetic rats [24,25], and in hole renal tissue specimens from patients with diabetic nephropathy [26].

It has been previously demonstrated that urinary AGT levels are increased in patients with chronic kidney diseases compared with control subjects [27]. In patients with type 1 or type 2 diabetes, we found that urinary AGT levels were significantly higher in pre-albuminuric patients with type 1 diabetes than in control subjects [28] and that ARBs reduced urinary AGT excretion in patients with type 2 diabetes [29]. Furthermore, we confirmed the results of the increased urinary AGT levels using rodent models such as streptozotocin-induced type 1 diabetic mice [23], db/db type 2 diabetic mice [30], and ZDF type 2 diabetic rats [31]. A recent study in rats indicated that urinary AGT originates from the AGT formed and secreted in the proximal tubules, and not from AGT in plasma [32]. Clearer evidence of AGT expression in human proximal tubules is, therefore, important for understanding the development and progression of diabetic nephropathy.

It has previously been reported that high glucose levels stimulate AGT expression via reactive oxygen species (ROS) generated from kidney cells [33]. We recently demonstrated that elevated ROS-associated augmentation of intrarenal AGT initiates the development of diabetic nephropathy in type 2 diabetic rats [24,25]. Moreover, overexpression of catalase in the kidney attenuates the augmented intrarenal AGT expression in diabetic mice [34]. However, clear evidence that RAS-related factors (especially AGT mRNA/protein) in the proximal tubules are increased in patients with diabetes is very scarce [35].

Therefore, this study was performed to demonstrate increased RAS-related factors (especially AGT mRNA/protein) in proximal tubules of patients with diabetes. We also addressed ROS-related factors in proximal tubules. Finally, we investigated the correlation between AGT expression levels and renal dysfunction in patients with type 2 diabetes.

## 2. Results and Discussion

### 2.1. Subjects’ Profiles and Laboratory Data

The demographics and the baseline laboratory data of the included subjects are summarized in Tables 1 and 2.

Baseline characteristics, including gender, age, height, body weight (BW), body mass index (BMI), systolic blood pressure (SBP), and diastolic blood pressure (DBP) are shown. There was a significant difference in the gender ratio, age, BMI, SBP, and DBP between the control and diabetic groups (Table 1).

Significant differences were also observed in the blood glucose (BG) levels, high density lipoprotein (HDL), urinary protein excretion (UPro), serum creatinine (Cr), creatinine clearance (Ccr), and estimated glomerular filtration rate (eGFR) between the control group and the diabetic group (Table 2). The results of the estimated eGFR (mL/min/1.73 m^2^) [36] were as follows: ≥90 in 5 patients; 60–89 in 5 patients; 30–59 in 15 patients; and 15–29 in 2 patients.

### 2.2. Histological Analysis (Masson’s Trichrome (MT)-Staining)

**Figure 1:**
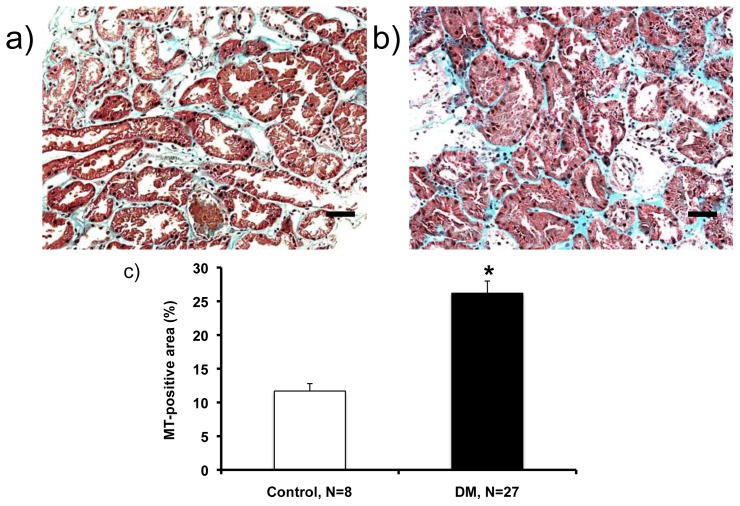
Masson’s trichrome (MT)-stained slides in control subjects and patients with diabetes. Compared with control subjects (**a**); the MT-stained area was larger in patients with diabetes (**b**); MT-positive area (%) is shown in (**c**). *p < 0.001 with control subjects. Magnification, ×200 (Scale bar, 100 μm).

The MT-stained area in the interstitium was significantly increased in patients with diabetes (26.2 ± 1.8; Figure 1b) compared with control subjects (11.7% ± 1.1%; Figure 1a) (Figure 1c; *p* = 0.0003).

### 2.3. Expression Levels of ROS- and RAS-Related Factors in the Kidneys of Control Subjects and Patients with Diabetes

**Figure 2:**
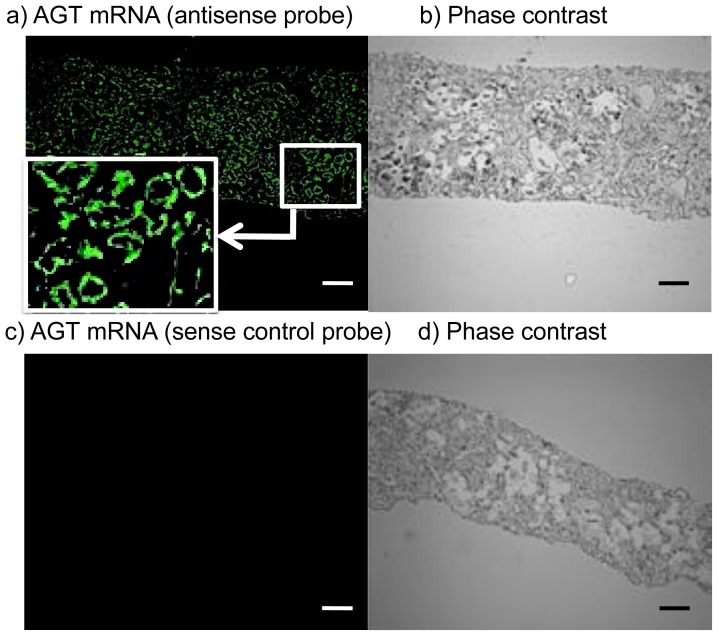
AGT mRNA expression in human kidney sections by fluorescence *in situ* hybridization (FISH). Sections were hybridized with antisense (**a**) the phase contrast image of the same area was shown in (**b**) or sense (**c**) the phase contrast image of the same area was shown in (**d**) probe. AGT, angiotensinogen. Magnification, ×100 (Scale bar, 50 μm).

Examples of whole images of AGT mRNA expression in human kidney sections using fluorescence *in situ* hybridization (FISH) are shown in Figure 2. We observed a strong staining signal using the designed locked nucleic acid (LNA)-antisense probe (Figure 2a,b) and no staining signal with the sense probe (Figure 2c,d).

**Figure 3:**
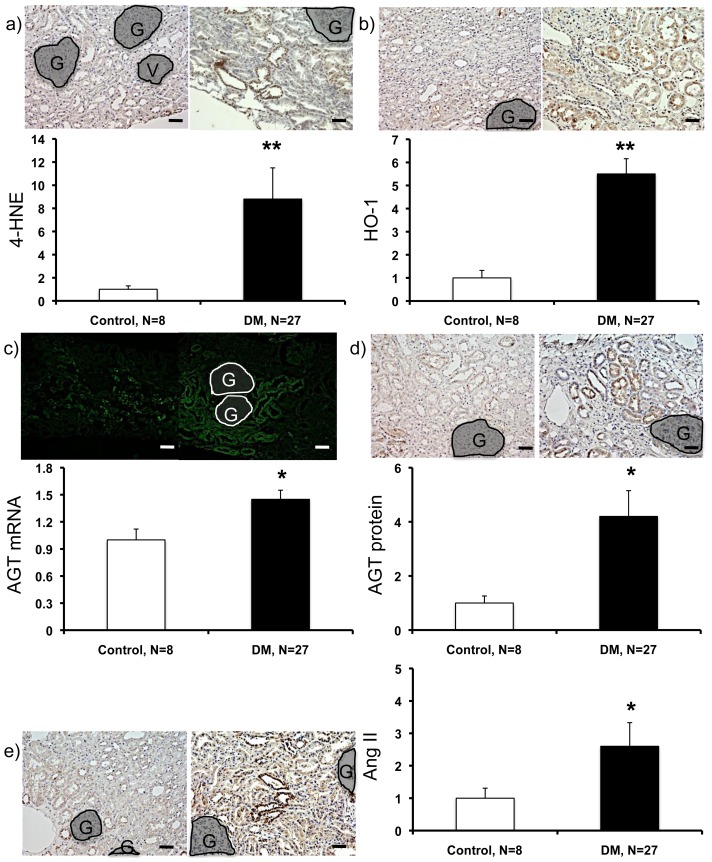
Expression levels of 4-HNE, HO-1, AGT mRNA, AGT protein, and Ang II. Data from 4-HNE (**a**), HO-1 (**b**), AGT mRNA (**c**), AGT protein (**d**), and Ang II (**e**) are shown. The areas of glomeruli and vessels were subtracted before analyzing the expression levels. 4-HNE, 4-hydroxy-2-nonenal; HO-1, hemeoxygenase-1; AGT, angiotensinogen; Ang II, angiotensin II; G, glomeruli; V, vessels. *p < 0.05 *vs*. control subjects, **p < 0.01 *vs*. control subjects. Magnification, ×200 (Scale bar, 100 μm).

The expression of ROS-related factors (4-hydroxy-2-nonenal (4-HNE) and heme oxygenase 1 (HO-1)) (Figure 3a,b) and RAS-related factors (AGT mRNA, AGT protein, and angiotensin II (Ang II)) (Figure 3c–e) were significantly greater in patients with type 2 diabetes compared with control subjects. The data are presented as fold increase compared with the levels in the control subjects.

### 2.4. Single-Regression Analysis for AGT mRNA Levels with Clinical Parameters in All Subjects (Control and Diabetes)

**Figure 4:**
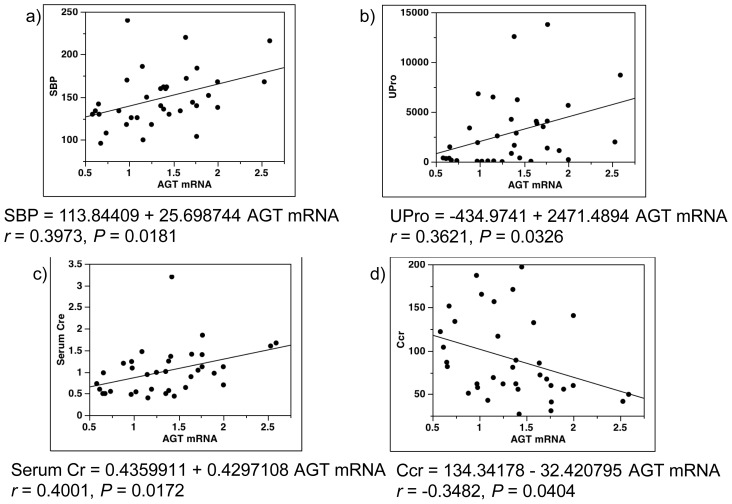

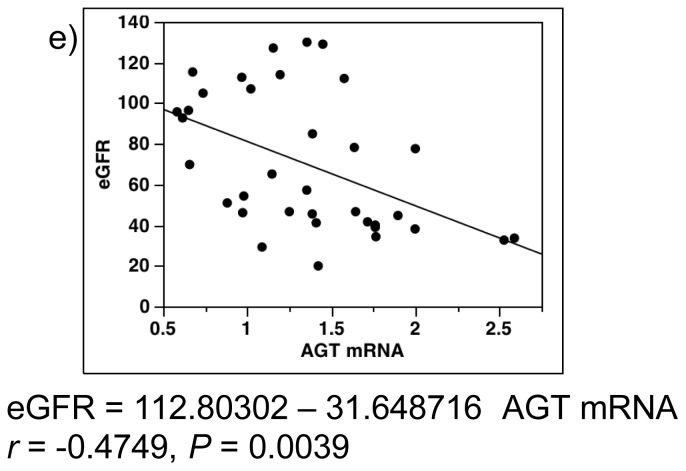
Single-regression analysis for AGT mRNA levels with clinical parameters. AGT mRNA levels were correlated with SBP (**a**); UPro (**b**); serum Cr (**c**); Ccr (**d**); and eGFR (**e**). AGT, angiotensinogen; SBP, systolic blood pressure; UPro, urinary protein excretion; Cr, creatinine; Ccr, creatinine clearance; eGFR, estimated glomerular filtration rate.

The results showed significant positive correlation of the AGT mRNA levels with SBP (Figure 4a), UPro (Figure 4b), and serum Cr (Figure 4c). Because two patients with diabetes had SBP > 220 mmHg (240 and 220 mmHg), we repeated the single-regression analysis of SBP and AGT mRNA after excluding these patients. The positive correlation between SBP and AGT mRNA remained statistically significant (SBP = 105.8065 + 36.594895 AGT mRNA, *r* = 0.5451, *p* = 0.0010). Although DBP tended to be positively correlated with AGT mRNA, the correlation was not statistically significant (*p* = 0.0609).

The results also showed significant negative correlation of AGT mRNA levels with Ccr (Figure 4d) and eGFR (Figure 4e). Because Ccr was high (Ccr > 180 mL/min/1.73 m^2^) in two subjects (197 mL/min/1.73 m^2^ in a patient with diabetes and 187 mL/min/1.73 m^2^ in a control subject), we repeated the single-regression analysis of Ccr and AGT mRNA after excluding these two subjects. In this analysis, the negative correlation between Ccr and AGT mRNA remained statistically significant (Ccr = 124.9022 − 39.018817 AGT mRNA, *r* = 0.3755, *p* = 0.0313).

### 2.5. Single-Regression Analysis for AGT mRNA Levels with the Expression Level of ROS- and RAS-Related Factors in All Subjects

**Figure 5:**
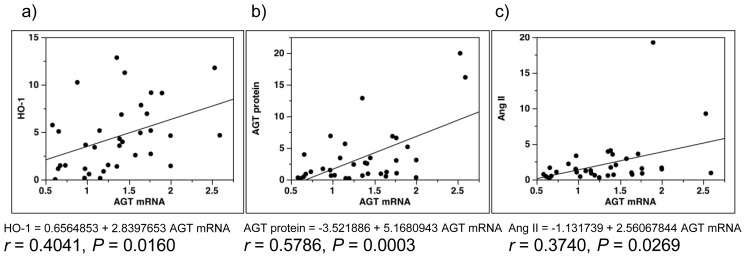
Single-regression analysis for AGT mRNA levels with the expression levels of ROS- and RAS-related factors. Single-regression analysis for AGT mRNA levels with HO-1 (**a**); AGT protein (**b**); and Ang II (**c**). AGT, angiotensinogen; ROS, reactive oxygen species; RAS, renin-angiotensin system; HO-1, hemeoxygenase-1; Ang II, angiotensin II.

Analyses showed significant positive correlation of AGT mRNA levels with HO-1 (Figure 5a), AGT protein (Figure 5b) and Ang II (Figure 5c).

### 2.6. Multiple-Regression Analysis for AGT Protein Levels in All Subjects

Factors with significant single correlations with AGT protein were adopted as explanatory variables in multiple-regression analysis. To reduce the impact of multicollinearity, we selected explanatory variables so that the mean sum of squares for the residual would be minimal in multiple-regression analysis. As a result, 4-HNE was excluded (Table 3).

**Figure 6:**
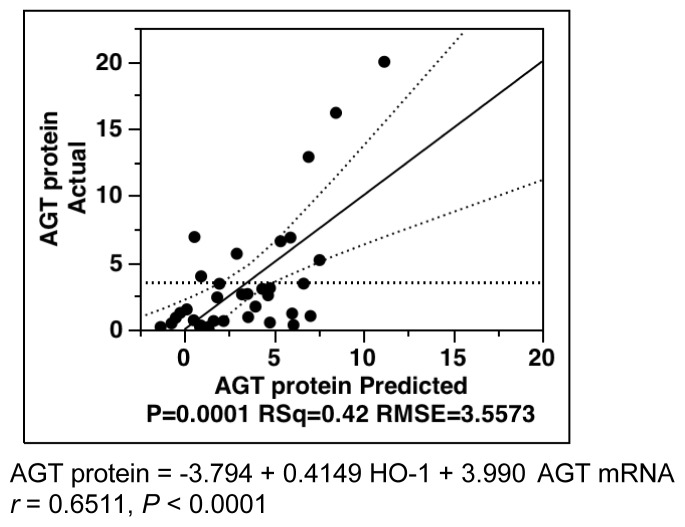
Multiple-regression analysis for AGT protein levels. Multiple-regression analysis for AGT protein level was evaluated using HO-1 and AGT mRNA parameters. These two parameters can account for over 40% of variation of AGT protein levels. AGT, angiotensinogen; HO-1, hemeoxygenase-1.

Using HO-1 and AGT mRNA parameters, the multiple-regression analysis was re-evaluated. As described in Figure 6, only two parameters can account for over 40% of the variation in the AGT protein levels (*r* = 0.6511; *R*^2^ = 0.4239; *p* = 0.0001).

### 2.7. Single-Regression Analysis for AGT mRNA Levels with eGFR and AGT Protein in Patients with Diabetes

**Figure 7:**
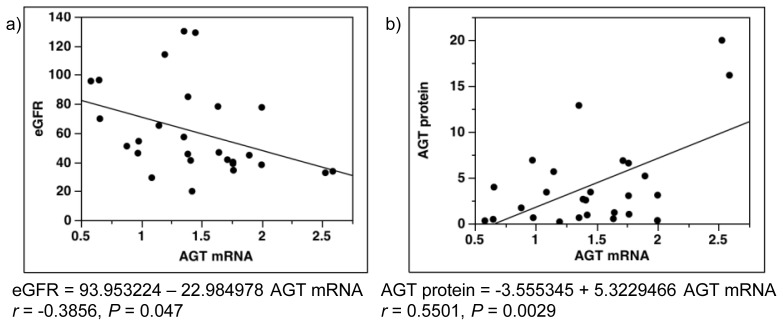
Single-regression analysis for AGT mRNA levels in patients with diabetes. AGT mRNA levels were correlated with eGFR (**a**) and AGT protein (**b**) in patients with diabetes. AGT, angiotensinogen; eGFR, estimated glomerular filtration rate.

The results showed significant negative correlation of AGT mRNA levels with eGFR (Figure 7a), and significant positive correlation with AGT protein (Figures 7b).

### 2.8. Comparison of AGT Expression in Patients with Diabetic Nephropathy Receiving Diabetes Medications (Insulin or Sulfonylurea (SU)) with Those Who Did Not Receive Such Medications

Because insulin and/or SU were being used to treat some patients with diabetic nephropathy, we compared the level of AGT expression observed in patients receiving diabetes medications (insulin or SU) with those who did not receive such medications. However, no differences were observed in AGT mRNA expression between patients receiving insulin or SU and those who did not (data not shown).

### 2.9. Discussion

Diabetic nephropathy is the most common cause of end-stage renal failure in developed countries, accounting for 45% of patients starting dialysis [37,38]. The use of drugs that block or interfere with the RAS has become common practice in treating patients with diabetes [9–11]. While the complicated and pleiotropic roles of an activated RAS in the pathogenesis of diabetic nephropathy have been well documented [3,39], the underlying mechanistic pathways have not been fully elucidated.

Urinary excretion rates of AGT provide a specific index of intrarenal RAS status [40–42]. It has been well demonstrated that urinary AGT is increased in diabetes in humans [28,29], mice [23,30], and rats [31]. In addition, enhanced intrarenal AGT mRNA and/or protein levels have been observed in diabetic mice [23,30] and rats [3,24,25]. In our previous trial [23], destruction of kidney tissue was not detected. The result suggests that an increase in angiotensinogen expression beyond a threshold level is involved in the development and progression of diabetic nephropathy. It goes without saying that it is impossible to address these mechanisms in a clinical study. We intend to clarify the significance and mechanisms involved in the increase in angiotensinogen expression in the development and progression of diabetic nephropathy in our future studies. A recent study in rats indicated that urinary AGT originates from the AGT formed and secreted in the proximal tubules, and not from AGT in plasma [32]. Therefore, in this study, we focused on AGT expression in proximal tubules. We have clearly demonstrated that intrarenal AGT mRNA and protein levels are increased in patients with diabetes compared with control subjects. Moreover, our data indicate that augmented intrarenal AGT mRNA expression parallels renal dysfunction, especially eGFR, in patients with diabetes. These data suggest that enhanced AGT expression in the kidney plays an important role in the pathogenesis of diabetic nephropathy.

While the mechanism of the enhanced intrarenal AGT in diabetes has not been fully elucidated, previous studies suggest a possible participation of the augmented ROS in this mechanism. The demonstration that high glucose levels augment *AGT* gene expression in rat immortalized renal proximal tubule cells provides a critical link between diabetes and AGT synthesis [20,33]. The rat *AGT* gene contains a putative insulin-responsive element in its promoter region [43,44], suggesting that glucose might regulate *AGT* gene expression. However, *in vivo* evidence, which demonstrates the linkage between ROS and AGT in the kidneys of type 2 diabetic rats, is insufficient to prove this, even though ROS-dependent intrarenal AGT activation plays an important role in hypertensive rats [45,46]. Previously, we reported increased intrarenal levels of AGT and the oxidative stress marker, urinary 8-isoprostane, in type 2 diabetic rats [24,25]. Elevated ROS levels may therefore synergize with augmentation of intrarenal AGT and initiate the development of diabetic nephropathy in type 2 diabetic rats. In this study, we have further clearly demonstrated that RAS-related factors (AGT mRNA, AGT protein, and Ang II) and ROS-related factors (4-HNE and HO-1) are increased in patients with diabetes compared with control subjects. Because this study corresponds to a single time point in the duration of diabetes in these patients, it may be difficult to address the sequential events of ROS- and RAS-activated processes for diabetic nephropathy. A recent study [34], however, provides supporting insight; the overexpression of catalase, a scavenger of hydrogen peroxide, attenuates intrarenal AGT expression in streptozotocin-induced diabetic mice. Furthermore, interstitial fibrosis and tubule apoptosis were attenuated by overexpressing catalase in proximal tubule cells from diabetic mice [47]. Taken together, these findings indicate that ROS-dependent intrarenal AGT activation appears to play an important role in the development of diabetic nephropathy.

We previously reported that urinary AGT is increased in patients with diabetes [27] and that treatment with ARBs attenuates the enhanced urinary AGT in patients with diabetes [29]. However, AGT is a protein, and proteinuria also increased in these patients. Therefore, it is possible that augmented urinary AGT in diabetes is a non-specific consequence of proteinuria. To rule out this possibility, we recently compared urinary AGT levels between juvenile patients with type 1 diabetes and age- and gender-matched control subjects. [28]. Neither urinary albumin levels nor urinary protein levels were found to be increased in patients with type 1 diabetes compared with the control subjects, suggesting that these patients were in the pre-microalbuminuric phase of diabetic nephropathy. Interestingly, the urinary AGT levels were significantly higher in patients with type 1 diabetes than in the control subjects. These data indicate that in patients with type 1 diabetes, the increase in urinary AGT levels precedes the increase in urinary albumin levels. Importantly, in the present study, we also showed that along with AGT protein levels, AGT mRNA levels are increased in the kidneys of patients with diabetes. Moreover, the augmented intrarenal AGT mRNA expression parallels renal dysfunction in patients with diabetes. These data may rule out the possibility of any casual coexistence between enhanced urinary AGT and enhanced proteinuria in patients with diabetes. Taken together, these results indicate that augmented urinary AGT excretion is not simply a non-specific consequence of proteinuria.

In the selection process in this study, women and men were recruited without any bias. Accordingly, there were some differences among the groups in terms of gender, age, BW, BMI, SBP, DBP, BG, HDL, UPro, serum Cr, Ccr, and eGFR (Tables 1 and 2), with an increased interstitial collagen-positive area; the pathological change seen in diabetic nephropathy. Most of these differences (BW, BMI, SBP, DBP, BG, HDL, UPro, serum Cr, Ccr, and eGFR) may have stemmed from well-recognized characteristics of patients with diabetic nephropathy [7,23–25,29,30,39,41]. We emphasize here that gender or age was not correlated with AGT mRNA levels in control subjects or in patients with diabetes in the single-regression analysis. In addition, we showed a correlation between AGT expression and eGFR in patients with only diabetic nephropathy (Figure 7). Therefore, AGT expression is increased, and is followed by the progression of diabetic nephropathy, indicating decreased eGFR. It seems unlikely that the above differences affected the final results reported herein.

Although we expected a relationship between AGT expression and high glucose, our data did not show the effect of clinical medication of insulin or SU (a hypoglycemic agent that enhances insulin secretion in pancreatic β-cells) in AGT mRNA expression in patients with diabetic nephropathy in a one-point sample collection study. A limitation of this study stems from the nature of the observational study using one-point sample collection. While these data establish a foundation that intrarenal levels of AGT mRNA and protein are increased in patients with diabetes, we recognize that a larger sample size is required to substantiate these observations.

In summary, we have demonstrated that levels of AGT mRNA and protein are significantly higher in patients with diabetes than in control subjects. Moreover, the augmented intrarenal AGT mRNA expression parallels renal dysfunction in patients with diabetes. These data suggest that intrarenal AGT activation plays an important role in the development of diabetic nephropathy. A prospective study to determine the relationship between the effect of RAS blockade on intrarenal/urinary AGT and cardiovascular events will be helpful in assessing the clinical significance of the decrease in intrarenal/urinary AGT with RAS blockade. Based on these findings, a randomized clinical trial has been projected to establish the clinical significance of the decrease in intrarenal/urinary AGT with RAS blockade.

## 3. Experimental Section

### 3.1. Study Design and Sample Collections

Patients were recruited in Osaka City General Hospital from new outpatients presenting with intermittent or persistent hematuria and/or proteinuria between May 2000 and February 2010, and who were later diagnosed with diabetic nephropathy or minor glomerular abnormality (MGA) by clinical and pathological findings. The study was a retrospective exploratory clinical study using samples of renal biopsies that were performed for detailed disease diagnosis. Sample collection is described below:

#### 3.1.1. Control Subjects

Renal biopsy was performed in response to the possibility of kidney disease. However, if the diagnosis was negative with no histopathological findings, the samples were used as controls. They were later diagnosed as MGA by clinical and pathological findings.

#### 3.1.2. Patients with Diabetic Nephropathy

When the physician strongly suspected that the kidney damage was caused by another disease such as chronic glomerulonephritis and not by diabetic nephropathy, a renal biopsy was necessary to differentiate between the diseases. A renal biopsy was also performed in patients with diabetes who presented with nephrosis, hematuria and/or treatment-resistance, because this is an effective method for confirming the diagnosis of nephritis. If other kidney diseases were excluded and a definite diagnosis of diabetic nephropathy was made by clinical and pathological findings, we used these samples as “patients with diabetic nephropathy”. In this study, patients who were taking any medications, who had previously received ACE inhibitors or ARBs, or had any cardiovascular event, were excluded. All patients who were diagnosed with diabetic nephropathy were immediately treated with the appropriate medical care (including administration of an ACE inhibitor and/or ARB).

Renal biopsied samples from 41 patients (29 with diabetic nephropathy and 12 with MGA) were obtained during this study period without any sampling bias. The experimental protocol of this retrospective exploratory clinical study was approved by the Institutional Review Board of Osaka City General Hospital. All patients provided written informed consent. All kidney samples were fixed in buffered-formalin immediately after removal and embedded in paraffin. Sequential 3-μm thick tissue sections were prepared by Mass Histology Service (Worcester, MA, USA) and then stained and examined. Two patients with diabetes and 4 subjects with MGA were excluded from further analyses because of the low quality of kidney samples. Therefore, the total number of participants in the study was 8 controls and 27 with type 2 diabetes. Blood and urine samples were obtained from participants within a week before the renal biopsy. Height, body weight, SBP, and DBP were also recorded on the same day. Blood pressure was measured as previously described [48]. Prior to taking the subjects’ blood pressure on their right arms, we allowed them to sit quietly for at least 5 min. We used an automated manometer (Omron, Kyoto, Japan) to make at least three blood pressure measurements 2 min apart. The mean blood pressure values were calculated from the last two systolic and diastolic pressure readings that fell within 5 mmHg of each other.

### 3.2. Measurements

Clinical laboratory data were measured in Osaka City General Hospital with an AU 400 Auto-analyzer (Olympus, Tokyo, Japan). The eGFR was calculated according to the formula established by the Japanese Society of Nephrology (JSN) in 2008 [eGFR (mL/min/1.73 m^2^) = 194 × Serum Creatine^−1.094^ × Age^−0.287^ × 0.739 (if the individual is woman)] [49].

### 3.3. FISH

Biotinylated LNA-modified DNA oligonucleotides (Exiqon, Woburn, MA, USA) were used for fluorescence *in situ* hybridization because of high hybridization, specificity and strong hybridization signal [18,50,51]. LNA probes for AGT were designed against antisense and sense sequences (antisense: 5′-biotin-CTT CCG CAT ACC CTT CTG CTG TAG-3′, *T*m = 80.0 °C; sense: 5′-biotin-CTA CAG CAG AAG GGT ATG CGG AAG-3′, *T*m = 81.0 °C). Sections were rehydrated by passage through 100%, 90%, 80%, 70%, and 40% ethanol baths and twice through phosphate buffered saline (PBS). To permeabilize tissues, sections were treated with 1 μg/mL proteinase K in PBS for 30 min at 37 °C and rinsed briefly in PBS containing 2 mg/mL glycine. Sections were post-fixed in 4% PFA for 20 min. Sections were pre-hybridized for 1 h, then hybridized for 18 h ((*T*m − 22)°C) with 10 nmol/L of either antisense or sense (for negative control) 5′-biotin-labeled LNA probes. The hybridization mix was composed of 1× salt, 50% formamide, 10% dextran sulfate, 1 mg/mL yeast RNA, and 1× Denhardt’s solution. After hybridization, sections were rinsed with wash solution (50% formamide, 1× SSC (Sigma-Aldrich, St. Louis, MO, USA), 0.1% Tween-20) for 15 min at 65 °C, followed by two 30-min washes with TNT wash buffer (0.1 mol/L Tris-HCl, pH 7.5, 15 mmol/L NaCl, 0.1% Tween-20) at room temperature. After blocking endogenous biotin (Invitrogen, Carlsbad, CA, USA), sections were incubated in TNB blocking buffer (0.1 mol/L Tris-HCl, pH 7.5, 15 mmol/L NaCl, 0.2% blocking reagent (Roche Diagnostics GmbH, Mannheim, Germany)) for 1 h at room temperature. Sections were subsequently incubated with 2 μg/mL Streptavidin, Alexa Fluor^®^ 488 conjugate (Molecular Probes, Eugene, OR, USA). Fifteen consecutive microscopic (immunofluorescence microscope; BX51, Olympus, Tokyo, Japan) fields in the selected tubule compartments (areas of glomeruli and vessels were excluded at the image analysis step) were examined, and the average intensity for each slide was calculated in a blind manner using Image-Pro plus software (Media Cybernetics, Bethesda, MD, USA). The histological sections were evaluated by three blinded pathologists (Evaluated by MU, TM, and YK). Afterwards, the expression data and the clinical data were collocated to perform statistical analyses without any bias.

### 3.4. Immunohistochemistry (IHC)

The intensities of AGT, Ang II, 4-HNE (a product of lipid peroxidation), and HO-1 (an inducible isoform in response to oxidative stress) were examined by IHC [18,52]. IHC was performed by an automated system (Autostainer; Dako, Carpinteria, CA, USA) and samples were counterstained with hematoxylin. The primary antibody against human AGT was obtained from Immuno-Biological Laboratories Co. Ltd. (Gunma, Japan) and the concentration for IHC was 1:400. The primary antibody against human Ang II was purchased from Phoenix (Belmont, CA, USA) and the concentration for IHC was 1:1000. The primary antibody against human 4-HNE, an unsaturated hydroxyalkenal that is produced by lipid peroxidation, was purchased from Japan Institute (Shizuoka, Japan) and the concentration for IHC was 1:100. The primary antibody against human HO-1, a microsomal enzyme that catalyzes the oxidation of heme, was purchased from Stressgen Bioreagents (San Diego, CA, USA) and the concentration for IHC was 1:3000. Immunoreactivity was quantitatively evaluated in a blind manner by a semi-automatic image analysis system using Image-Pro plus software (Media Cybernetics, San Diego, MD, USA) as previously described [52]. For each slide, 20 consecutive microscopic fields of selected tubule compartments (as described in Section 3.3) were examined and the averaged intensities were obtained. The histological sections were evaluated by three blinded pathologists. Afterwards, the expression data and the clinical data were collocated to perform statistical analyses without any bias.

### 3.5. Histological Analysis

For histological analysis, MT-staining on formalin-fixed, paraffin-embedded renal sections was performed by an out-sourcing company (Kyodo Byori Inc., Hyogo, Japan). The extent of the interstitial collagen-positive area was evaluated quantitatively by an automated image analysis method, which determined the area occupied by interstitium tissue staining positive for collagen in the MT-stained section. Quantification was performed semi-automatically in a blind manner using Image-Pro plus software [52].

### 3.6. Statistical Analysis

To analyze clinical data, statistical significance between any two groups was analyzed by Chi-square tests or unpaired *t*-tests. Data for expression levels were evaluated using the Wilcoxon signed-rank test. Correlations were determined using the Pearson correlation coefficients. Standard least-squares method was used for multiple-regression analysis. All data are presented as the means ± SEM. Values were considered significant at *p* < 0.05. All computations, including data management and statistical analyses, were performed with JMP software (SAS Institute, Cary, NC, USA).

## 4. Conclusions

The expression of AGT, Ang II, 4-HNE, and HO-1 is greater in patients with diabetes than in control subjects. The augmented intrarenal AGT mRNA expression parallels renal dysfunction in patients with diabetes. The activated oxidative stress/AGT/RAS axis is important in the pathogenesis of diabetic nephropathy. These results support the clinical significance of the reduction of intrarenal/urinary AGT by RAS blockade in diabetic nephropathy.

## Figures and Tables

**Table 1:** Subject profiles.

Parameters	Control subjects (*N* = 8)	Patients with type 2 diabetes (*N* = 27)	*p*-Value	χ^2^
Gender, W/M	7/1	9/18*	0.0069	7.296
Age, y	37.0 ± 3.3	54.6 ± 2.7*	0.0020	-
Height, cm	161.1 ± 2.9	161.2 ± 1.8	0.9885	-
BW, kg	51.5 ± 5.0	65.4 ± 2.2*	0.0071	-
BMI	19.6 ± 1.1	25.2 ± 0.8*	0.0013	-
SBP, mmHg	116.8 ± 5.1	157.9 ± 6.0*	0.0010	-
DBP, mmHg	69.3 ± 3.1	88.5 ± 2.7*	0.0010	-

W indicates women; M, men; BW, body weight; BMI, body mass index; SBP, systolic blood pressure; DBP, diastolic blood pressure,

* *p* < 0.05 *vs.* control subjects.

**Table 2:** Laboratory data.

Parameters	Control subjects (*N* = 8)	Patients with type 2 diabetes (*N* = 27)	*p*-Value
BG, mg/dL	91.7 ± 6.2	168.1 ± 11.7 *	0.0027
HbA1c, %	(N/A)	7.5 ± 0.4	(N/A)
T-Cho, mg/dL	195.0 ± 19.0	237.0 ± 12.9	0.1141
TG, mg/dL	136.8 ± 52.7	243.9 ± 27.4	0.0732
HDL, mg/dL	68.7 ± 6.9	51.4 ± 2.2 *	0.0036
LDL, mg/dL	104.6 ± 14.3	135.4 ± 11.4	0.2014
UNA, g/day	2.3 ± 0.2	2.5 ± 0.2	0.4555
UCl, g/day	3.2 ± 0.4	3.3 ± 0.3	0.8884
UNaCl, g/day	5.5 ± 0.6	5.8 ± 0.5	0.6877
UPro, mg/day	105.6 ± 34.2	3727.1 ± 685.1 *	0.0075
Serum Cr, mg/dL	0.6 ± 0.1	1.1 ± 0.1 *	0.0095
Ccr, mL/min/1.73 m^2^	136.7 ± 13.8	76.9 ± 7.9 *	0.0009
eGFR, mL/min/1.73 m^2^	102.3 ± 8.7	60.6 ± 5.9 *	0.0012

HbA1c was measured according to the method of the Japan Diabetes Society (JDS) unit. BG indicates blood glucose; HbA1c, hemoglobin A1c; T-Cho, total cholesterol; TG, triglyceride; HDL, high density lipoprotein; LDL, low density lipoprotein; UNA, urinary sodium excretion; UCl, urinary chloride excretion; UNaCl, urinary sodium chloride excretion; UPro, urinary protein excretion; Cr, creatinine; Ccr, creatinine clearance; eGFR, estimated glomerular filtration rate.

* *p* < 0.05 *vs.* control subjects.

**Table 3:** Multiple-regression analysis by the Stepwise method for AGT protein.

Parameters	Estimate	SE	*T* Ratio	*p* Value
Intercept	−3.7943	1.7293	−2.19	0.0356 *
4-HNE	−0.0519	0.0496	−1.05	0.3029
HO-1	0.4149	0.1865	2.23	0.0332 *
AGT mRNA	3.9899	1.3102	3.05	0.0046 *

4-HNE indicates 4-hydroxy-2-nonenal; HO-1, hemeoxygenase-1; AGT, angiotensinogen.

* *p* < 0.05.
